# Left Atrial Appendage Occlusion Versus Direct Oral Anticoagulation in Atrial Fibrillation Patients at Very High Risk of Stroke: A Budget Impact Analysis in Italy

**DOI:** 10.3390/jcm15103687

**Published:** 2026-05-11

**Authors:** Michele Magnocavallo, Ahmad Awada, Guccio Vagnarelli, Pietro Rossi, Ilaria Giabbani, Elisa Vireca, Giampaolo Vetta, Alexandre Almorad, Gaetano Chiricolo, Antonio Sorgente, Carlo de Asmundis, Gian-Battista Chierchia, Stefano Bianchi, Andrea Natale, Domenico Giovanni Della Rocca

**Affiliations:** 1 Arrhythmology Unit, Isola Tiberina-Gemelli Isola Hospital, 00186 Rome, Italy; michelefg91@gmail.com (M.M.); 2 Department of Cardiology, CHU Brugmann, Université Libre de Bruxelles, 1020 Brussels, Belgium; 3 Boston Scientific, 20134 Milan, Italy; guccio.vagnarelli@bsci.com (G.V.); ilaria.giabbani@bsci.com (I.G.); 4 Boston Scientific, 1831 Diegem, Belgium; 5 Heart Rhythm Management Centre, Postgraduate Program in Cardiac Electrophysiology and Pacing, Universitair Ziekenhuis Brussel—Vrije Universiteit Brussel, European Reference Networks Guard-Heart, 1090 Brussels, Belgium; 6 Department of Biomedicine and Prevention, Division of Cardiology, University of Tor Vergata, 00133 Rome, Italy; 7 Texas Cardiac Arrhythmia Institute, St. David’s Medical Center, Austin, TX 78705, USA; 8 Interventional Electrophysiology, Scripps Clinic, San Diego, CA 92037, USA; 9 Metro Health Medical Center, Case Western Reserve University School of Medicine, Cleveland, OH 44109, USA

**Keywords:** atrial fibrillation, stroke, watchman, left atrial appendage, direct oral anticoagulant

## Abstract

**Background:** Left atrial appendage occlusion (LAAO) requires a significant upfront investment, which is in contrast with the more gradual, long-term costs of direct oral anticoagulants (DOACs). **Objective:** We performed a budget impact analysis exploring the financial impact of increasing the number of LAAO procedures in a high-stroke-risk population over a 10-year time horizon from the perspective of the healthcare providers under the Italian National Healthcare Service. **Methods:** Two alternative scenarios simulating an increased uptake of the LAAO therapy were compared to the estimated volume of LAAO procedures performed (baseline scenario: 1341 procedures): (1) Alternative Scenario I (3314 procedures) based on the level of penetration observed in the Italian region performing the highest rate of LAAO procedures; (2) Alternative Scenario II (7672 procedures): LAAO therapy uptake set to attain 5% of the estimated target population. Clinical data were extracted from a propensity-matched, multicenter cohort of 554 AF patients at a very high thromboembolic risk profile (CHA_2_DS_2_-VASc score ≥ 5) treated with LAAO or DOACs. **Results:** Cumulative cost savings in Alternative Scenario I were around €4.9 million compared to the baseline. When comparing Scenario II to the baseline scenario, savings added up to €15.8 million over 10 years. The break-even point occurred between the seventh and eighth years. Cost savings were observed even in the instance that all DOAC prices would decrease as generics became available. **Conclusions:** The widespread use of LAAO therapies in a population of AF patients at very high stroke risk may yield substantial long-term benefits, as the initial investment in the LAAO procedure and device would be counterbalanced within 8 years.

## 1. Introduction

Atrial fibrillation (AF) affects 33 million people worldwide and 11 million in Europe as of 2020 [1,2]. This condition significantly increases the risk of stroke, heart failure, and other cardiovascular complications, thereby contributing to substantial morbidity, mortality, and economic burden on healthcare systems.

AF patients have a ~five-fold higher risk of experiencing a stroke than those without AF; furthermore, AF-related strokes tend to be more severe, with higher rates of disability and a mortality rate of up to 25% within the first 30 days [3].

As its prevalence is projected to double by 2030, the healthcare and economic impact of AF is expected to concurrently rise [4]. In 2017, the total cost of stroke across the European Union reached €60 billion, with Italy’s share totaling approximately €7 billion. This amount represents approximately 2% of the nation’s healthcare spending [5].

The increased stroke severity and the associated healthcare costs resulting from long-term disability underline a critical need for cost-effective stroke prevention strategies among AF patients [6]. Long-term direct oral anticoagulation (DOAC) is the mainstay therapy for thromboembolic (TE) prevention and is currently preferred over vitamin K antagonists due to a more favorable safety profile [6,7]. Nonetheless, a large share of AF patients eligible for long-term TE prophylaxis either do not receive an appropriate pharmacological therapy or have a contraindication for long-term DOAC due to a history of major bleeding complications [7,8]. Non-adherence, under- or over-dosing are the main factors precluding an adequate long-term stroke prevention strategy and potentially predisposing patients to serious adverse events [9,10].

Percutaneous left atrial appendage occlusion (LAAO) is an effective alternative to long-term oral anticoagulation, especially for patients who are ineligible for long-term DOAC or are prone to major bleeding [11,12,13,14]. Although no differences were observed between these two strategies in terms of cardioembolic events [15], the primary benefit of LAAO is its reduced bleeding risk, especially in patients with a high burden of cardiovascular disease [11,13,16,17].

Budget impact analysis is an essential component of a comprehensive assessment of innovative healthcare interventions, typically required for reimbursement evaluation purposes [18]. The present budget impact analysis explored the financial impact of increasing the number of LAAO procedures in non-valvular AF patients with CHA_2_DS_2_-VASc score ≥ 5 currently receiving DOAC therapy (our reference patients’ cohort) over a 10-year time horizon and from the perspective of the Italian National Healthcare Service.

## 2. Methods

### 2.1. Budget Impact Model

The impact of the two treatment options (LAAO vs. DOAC) on disease progression (incidence of adverse events) was modeled using clinical data from a previous study comparing the safety and efficacy of LAAO vs. DOACs in patients with AF at very high stroke risk (CHA_2_DS_2_-VASc score ≥ 5). The net budget impact was calculated by comparing the projected costs for each comparator: potential budget savings or gains after 10 years were expressed as the difference in the healthcare costs incurred to manage the reference patient cohort of very high patients between a baseline scenario, which mirrors the estimated current volume of LAAO procedures in Italy, and two alternative scenarios simulating increased LAAO adoption within the same cohort. All participants gave written informed consent for each interventional procedure and data collection. The data underlying this article will be shared on reasonable request to the corresponding author.

### 2.2. Clinical Data

Clinical data were prospectively collected between January 2017 and January 2020 and were extracted from a multicentre perspective Institutional Review Board-approved database in which all baseline characteristics, echocardiographic parameters, drug therapy, and follow-up data of AF patients from six enrolling centers. From an initial cohort including 1053 LAAO and 1328 DOAC patients, AF patients at high TE risk (CHA_2_DS_2_-VASc score ≥ 5) were selected. To attenuate the imbalance in covariates between the two groups, propensity score matching was performed, resulting in a matched population with 277 CHA_2_DS_2_-VASc score ≥ 5 patients per group [13]. For the purpose of this study, only patients with a minimum follow-up of 12 months were included in the final analysis. In the LAAO group, percutaneous occlusion was performed via a Watchman 2.5 or Watchman FLX^™^ device (Boston Scientific Corporation, Marlborough, MA, USA); patients who received other devices or surgical occlusion of the left atrial appendage were excluded from our analysis. The device implantation technique has been previously described elsewhere [16,19,20]. Eligibility criteria included ≥18 years of age with nonvalvular AF who were unsuitable for long-term DOAC (previous major bleeding or contraindication for oral anticoagulation) and a CHA_2_DS_2_-VASc score of ≥3. In the DOAC group, patients were prescribed an appropriate dose of anticoagulation according to the latest guidelines on dose selection criteria [6].

### 2.3. Epidemiology and Patient Funnel

A funnel approach was used to estimate the reference cohort of AF patients at high risk of stroke (CHA_2_DS_2_-VASc score ≥ 5) currently undergoing pharmacological treatment with DOAC in Italy (Table 1). Based on demographic and epidemiologic data, stroke risk assessment (CHA_2_DS_2_-VASc score) and the most recent data on medical prescriptions, a total of 153,439 patients were estimated for Italy in 2025 (Table 1).

### 2.4. Scenario Analysis

Two alternative scenarios were compared to a baseline scenario of 1341 LAAOs, reflecting the estimated volume of procedures performed in patients with a CHA_2_DS_2_-VASc score ≥ 5 in Italy in 2024. Of the 2539 procedures performed in 2024, according to the Italian Society of Interventional Cardiology (GISE) registry [28], we estimated that around 52.8% were carried out in high-risk patients [15].

The two alternative scenarios simulated an increased uptake of the LAAO therapy within the reference cohort of DOAC patients:

(a) *Alternative Scenario I (3314 LAAO procedures):* This estimate was derived by extrapolating the penetration rate observed in Sicily—the region with the highest number of LAAO procedures per population in 2024 (10.6 per 100,000 inhabitants)—to the entire Italian population [28], and then restricting the estimate to high-risk patients [15].
(b) *Alternative Scenario II (7672 LAAO procedures):* LAAO therapy uptake was set to attain 5% of the reference patients’ cohort.

In both scenarios, continued DOAC therapy was presumed for the remaining patients in the DOAC target population for the whole 10-year period analyzed.

### 2.5. Model Structure and Assumptions

**Figure 1:**
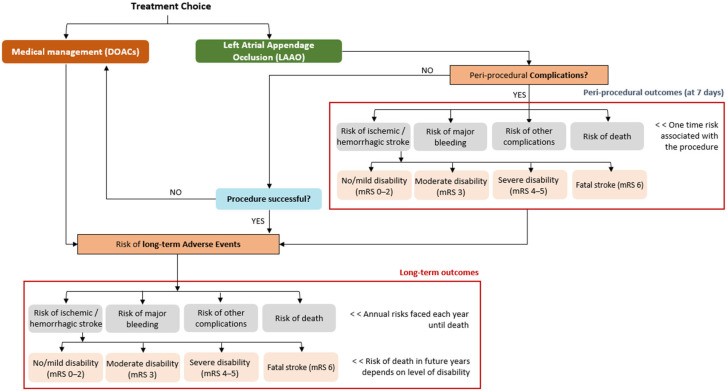
Diagram of model flow. DOAC: direct oral anticoagulation; LAAO: left atrial appendage occlusion.

The budget impact model was developed in Microsoft Excel (Microsoft 2023 Excel, Redmond, WA, USA; Microsoft Corporation). Patients entered the model at the time of the LAAO procedure or initiation of DOAC (Figure 1). Patients undergoing LAAO were subject to procedure-related events as reported in the Supplemental Material. After LAAO, patients were discharged under short-term therapy (before 6 months) with full-dose DOAC plus aspirin [n = 236, (85.2%)] or full-dose DOAC alone [n = 41, (14.8%)]. After 12 months post/implant, we assumed all patients discontinued DOACs but received aspirin until the end of the model time horizon.

In the DOAC cohort, the type and dosage of DOACs were the same as those reported in the previous study [13]. Patients were assumed to remain on DOAC therapy for the entire model time horizon. Over a 10-year time horizon commencing in 2025, patients in both cohorts were considered to have a yearly risk of adverse events. Adverse events are not mutually exclusive, meaning each patient can experience more than one event per year. Event rates were expressed as annualized rates (i.e., incidence rate per 100 patient-years) and were assumed to remain constant throughout the 10-year duration of the analysis. The only exception was for the rate of bleeding in the LAAO cohort, which is known to exhibit a significant reduction 1 year post-implant, as a result of adjustments or discontinuation in antithrombotic therapy [13,29,30,31]. Event rates were retrieved from the study, which informed the analysis [13]. For the analysis, non-clinically relevant minor bleedings were not considered, as they do not represent a direct economic burden for healthcare providers. Definitions of clinical endpoints were reported in the Supplemental Materials. Disability severity for each treatment arm was extracted from the original dataset, categorizing post-stroke patients into three groups based on the modified Rankin scale (mRS): no or mild disability, moderate disability, and severe disability [32].

### 2.6. Costs

The cost inputs applied in the model are presented in Supplemental Table S1. The estimated cost of the LAAO procedure includes the cost of the WATCHMAN FLX^™^ device (Boston Scientific Corporation, Marlborough, MA, USA), the cost of the cath lab and personnel (assuming a procedural duration of 60 min [33], n.4 physicians and n.3 nurses employed) and the cost of hospitalization (assuming an average length of stay of 1.5 days) [34]. Actual costs incurred by hospitals for peri-procedural complications and adverse events were retrieved from the literature [35,36,37] (Supplemental Table S1). For the other events, the Italian DRG national reference tariff was used as the best proxy of costs incurred by hospitals, as more accurate micro-costing investigations were not available. Note that the same unit costs were assumed for clinical events regardless of whether they occurred during the procedure (peri-procedural complications) or in the post-procedural phase (adverse events).

Information on drug costs and Defined Daily Doses (DDD) was obtained from Codifa, the Italian drug information system, and from resolutions issued by the Italian Medicines Agency (AIFA—Agenzia Italiana del Farmaco). The ex-factory price was applied for all drugs considered (i.e., net of distribution margins), and the generics price was applied when available in the market.

All monetary values used in this analysis were adjusted for the Consumer Price Index for Families of Workers and Employees and reported in 2025 Euros, using a tool provided by the Italian National Institute of Statistics (ISTAT) [38]. Discounting was not applied, as recommended by ISPOR Guidelines [18].

## 3. Results

### 3.1. Baseline Characteristics and Clinical Data

Among 2381 patients, propensity score matching identified 277 CHA_2_DS_2_-VASc score ≥ 5 patients per group with a minimum follow-up of 12 months. Demographic and clinical characteristics of the matched populations are reported in the Supplemental Table S2. In the DOAC group, 211 (76.2%) patients were on direct factor Xa inhibitors [Rivaroxaban: 59 (21.3%); Apixaban: 135 (48.8%); Edoxaban: 17 (6.1%)] and the remaining 66 (23.8%) on a direct oral thrombin inhibitor.

In the LAAO group, the periprocedural overall adverse event rate was 2.9% (n = 8); major complications occurred in 5 (1.8%) patients [3 pericardial effusions requiring surgical/percutaneous drainage, one transient ischemic attack, and one clinically relevant bleeding] (Supplemental Table S3).

The mean overall follow-up was 25 ± 6 months; no difference was reported in the incidence rate of TE events [4.1 events/100 patient-years (py) in DOAC group vs. 3.2 events/100 py in LAAO group; log-rank *p*-value = 0.63] (Supplemental Table S4).

The incidence of major bleeding was significantly higher with DOACs (major bleeding: 2.9 events/100 py vs. 1.1 events/100 py with LAAO, *p*-value = 0.03) (Supplemental Table S4). The rate of clinically relevant minor bleeding for LAAO in the study dataset is 2.9 events per 100 py (first year) and 1.1 events per 100 py (second year). Major bleedings for LAAO were 1.4 events per 100 py (first year) and 0.4 events per 100 py (second year). To reflect this, a reduction of 62.1% for clinically relevant minor bleeding and 71.4% for major bleeding was applied after the first year.

### 3.2. Scenario Analysis

The total cost of managing the AF target population when extending LAAO to 3314 patients (Alternative Scenario I) was approximately €2229 million. Compared to approximately €2234 million in the baseline scenario, this resulted in cumulative cost savings of around €4.9 million over the 10-year model time horizon.

When comparing scenario II (total cost of approximately €2218 million) to the baseline scenario, savings added up to around €15.8 million over 10 years. According to the budget impact analysis performed, each additional patient treated with LAAO produced a saving of €2489 for healthcare providers over a 10-year time horizon.

Table 2 provides a detailed breakdown of cumulative cost savings by cost category for all assessed scenarios. In both scenarios, cost savings were mainly driven by reduced incidence of major bleedings, strokes, and post-stroke disability due to increased LAAO therapy uptake and post-implant DOAC discontinuation.

**Figure 2:**
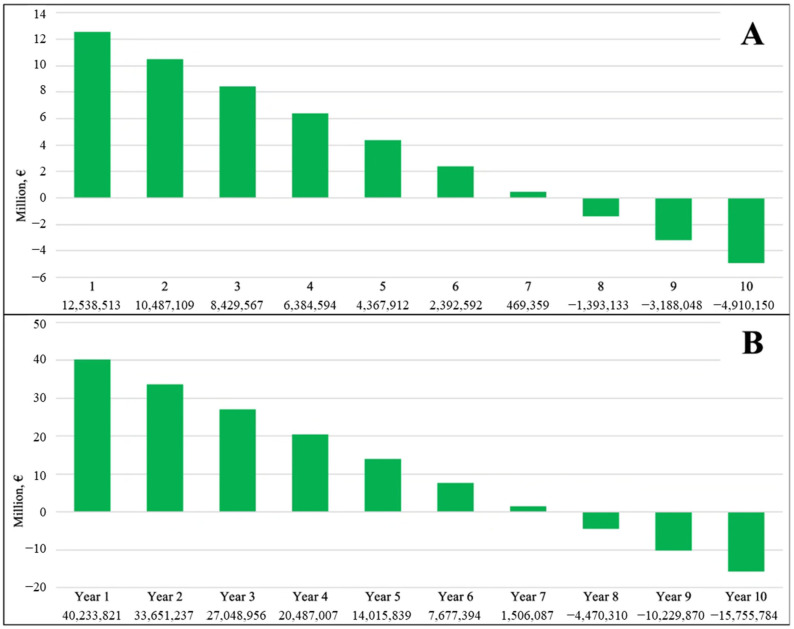
Ten-year national cumulative budget impact of increasing adoption of LAAO from the current baseline (1341) to Scenario I (3314) (**A**) and from the current baseline (1341) to Scenario II (7672) (**B**). LAAO: left atrial appendage occlusion.

Cumulative costs over time for Alternative Scenarios I and II, relative to the baseline scenario, are presented in Figure 2, enabling assessment of cost trajectories and the timing of the *break-even point*. The *break-even point* of the budget impact analysis, at which the total costs associated with Alternative Scenario I matched those of the baseline scenario, occurred between the seventh and eighth year (Figure 2A), when cost savings offset the initial investments required to sustain LAAO therapy expansion. Similarly, when investigating scenario II vs. baseline, the break-even point fell between the seventh and eighth year (Figure 2B).

Based on the budget impact analysis, patients in the current scenario would suffer 19,472 strokes and 31,196 major bleedings. Expanding LAAO therapy to 3314 patients (scenario I) might prevent 41 ischemic strokes, resulting in savings of €267,912 over 10 years and 694 major bleedings, which might save an additional €2.5 million in healthcare costs (Table 3). Furthermore, by preventing stroke-related disability, an additional €6.89 million in long-term disability management costs could be avoided (Table 2). The model estimated benefits to grow even larger if 5% of the reference population received LAAO (scenario II). This strategy would potentially prevent 130 strokes and 1040 major bleedings compared to the current scenario, resulting in savings of €859,681 and €8,030,780, respectively, for the Italian National Healthcare Service through avoided hospitalizations (Table 3), along with an estimated €22.1 million reduction in long-term disability-related healthcare expenditures.

### 3.3. Sensitivity Analysis

One-way sensitivity analysis was conducted by varying all model parameters within their 95% confidence intervals (or ±10% of the base case value) and assessing the effect on the 10-year cumulative budget impact for both Scenario I and Scenario II, compared to baseline.

**Figure 3:**
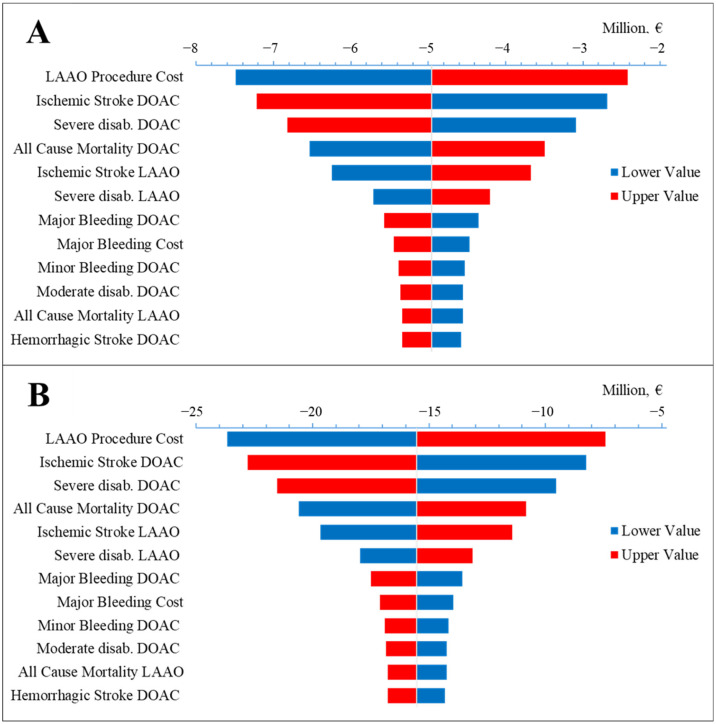
Tornado plot of sensitivity analysis. The x-axis represents the cost savings in Alternative Scenario I versus baseline at 10 years (**A**) and in Alternative Scenario II versus baseline at 10 years (**B**). DOAC: direct oral anticoagulation; LAAO: left atrial appendage occlusion.

The most impactful costs were those of the LAAO procedure and the cost of an episode of major bleeding (Figure 3). Clinical variables yielding the largest variability were ischemic stroke (for both LAAO and DOAC patients), major bleeding and minor bleeding (for DOAC patients), and haemorrhagic stroke (for DOAC patients) (Figure 3). Furthermore, the sensitivity analysis highlights the rate of severe disabling strokes among both DOAC- and LAAO-treated patients as a key factor substantially affecting model outcomes. Finally, the model exhibited notable responsiveness to variations in all-cause mortality rates within both treatment arms. All parameter variations impacted the economic results in the expected direction; in all simulations, the alternative Scenario maintained the cost-saving option at 10 years. The complete list of parameters whose impact on the 10-year cumulative budget was greater than 5% in the alternative scenario analysis can be found in Supplemental Figures S1 and S2.

We then evaluated the responsiveness of the model to drug pricing assumptions. Given that Dabigatran and Rivaroxaban are already available as generics on the Italian market, their current market prices were directly incorporated into the model (Supplemental Table S1). To simulate future price reductions for the remaining two DOACs (Apixaban and Edoxaban), we ran a scenario in which their prices were aligned with those of the generics, based on the assumption that their original prices were comparable and would follow a similar downward trend upon generic entry. Under these assumptions, Scenario II continued to yield approximately €4.6 million in savings over 10 years.

## 4. Discussion

Herein, we performed a budget impact analysis aimed at assessing the 10-year financial impact of increasing the number of LAAOs in very high-risk patients, from the perspective of healthcare providers under the Italian Healthcare Service. We started from a yearly total volume of 1341 LAAO procedures that was adopted as baseline reference according to the latest data from GISE [28]. We then simulated an increased uptake of LAAO procedures following two alternative scenarios, corresponding to the level of penetration observed in the Italian region, which currently has the highest number of LAAO procedures per population size (Scenario I) and to 5% of the estimated reference AF population (Scenario II).

This analysis supports the idea that increased adoption of LAAO over DOACs could yield substantial cost savings for patients with a high risk of TE events and bleeding complications. Specifically, our findings suggest that both alternative scenarios I and II may contribute to significant cost savings (€4.9 million and €15.8 million, respectively) over a 10-year model time horizon. Of note, the break-even point, such as the time point when cost savings offset the initial investments required to sustain LAAO therapy expansion, would occur between the seventh and eighth year in both alternative scenarios. Furthermore, the sensitivity analysis suggests that cost savings might be observed even in the instance that all DOAC prices would decrease as generics become available.

DOACs have become the preferred option over traditional vitamin K antagonists for most non-valvular AF patients. However, a significant portion of high-risk AF patients remain inadequately treated, leading to increased health risks. Conditions like active bleeding disorders, advanced liver or kidney disease, and a history of major or recurrent minor bleeding events may limit the use of DOACs, placing patients in a complex situation where they concomitantly face elevated stroke and bleeding risk [10,39,40,41]. In such cases, clinicians must weigh the risks of bleeding against the benefits of stroke prevention, often leading to either lower-than-recommended doses or withholding anticoagulation altogether. The ultimate result is that many high-risk AF patients are undertreated due to contraindications for long-term anticoagulation and face increased risks associated with non-adherence or dosing issues. A recent analysis of the prospective Global Anticoagulant Registry in the Field-Atrial Fibrillation (GARFIELD-AF) showed that approximately one fourth (27.1%) of AF patients with an indication to standard-dose DOAC are either underdosed (23.2%) or overdosed (3.8%) [42]. No recommended dosing was associated with a 24% higher risk of all-cause mortality, which was mostly related to higher rates of cardiovascular death, mainly myocardial infarction and congestive heart failure. Studies consistently demonstrated worse clinical outcomes compared to the recommended dosage group [43,44,45].

LAAO is an effective alternative, especially for patients with a high comorbid burden or a history of TE and/or major bleeding. Specifically, previous reports have demonstrated that LAAO is at least non-inferior to DOACs for stroke prophylaxis, and is associated with a significant reduction in major and minor bleeding events in the mid- to long-term [13,15,46,47,48]. Treating AF patients with LAAO requires a significant upfront investment, which is in contrast with the more gradual costs of DOACs that are spread over time. In Italy, spending on DOACs accounts for approximately 2% of the total expenditures by the Italian National Healthcare System [49]. Given the increasing prevalence of AF [4], it is essential to explore alternative therapies to guarantee the sustainability of the healthcare system. Another key factor to take into consideration is that technological advancements have led to a wider adoption of catheter ablation [50,51,52]. As a result of the development of novel non-thermal energy-based technologies featuring high cardiac tissue selectivity, namely pulsed electric field, catheter ablation is now more efficient and effective, as well as safer [52,53,54]. Current data indicate that catheter ablation significantly reduces the burden of AF compared to medical therapies [10]. In light of this, LAAO might become a first-line post-ablation strategy as it minimizes long-term anticoagulation burden, thereby balancing stroke prevention with safety and quality of life. The OPTION trial is a randomized clinical study designed to evaluate the effectiveness and safety of catheter ablation combined with LAAO versus catheter ablation combined with DOAC therapy [55]. The results showed superiority of LAAO for non-procedural major and clinically relevant non-major bleedings and non-inferiority for a composite of death from any cause, stroke, or systemic embolism at 36 months.

These are just some of the reasons why policymakers should carefully assess optimal treatment options for AF patients with different risk profiles, not solely from a budgetary perspective, but also to respect patient preferences and enhance quality of life in appropriately selected cases.

The potential of LAAO to yield budget neutrality within a clinically reasonable timeframe further strengthens the case for healthcare policy changes towards higher adoption rates of LAAO. The results appear to be in line with the already published evidence. Previous budget impact analyses conducted in Europe [36,56,57] suggested that increasing LAAO penetration vs. warfarin and DOACs would yield a positive economic effect on the budget over a similar time horizon. Similarly, cost-effectiveness analyses, mainly from North America [58,59,60,61,62,63,64,65], have proven LAAO to be a cost-effective alternative to the standard of care.

As such, an initial investment to sustain LAAO expansion would be counterbalanced by the increased healthcare costs secondary to the adverse event burden associated with DOACs within a 7–8-year timeframe. While the model assumes a rapid increase in therapy adoption, it is important to note that achieving such adoption rates in real-world settings presents significant challenges. Addressing these challenges would require overcoming system-level constraints, including limitations in hospital capacity and referral pathways. Furthermore, disparities in financial investment across regions and gaps in implant expertise between high- and low-volume centers need to be addressed to ensure equitable access to a safe and effective treatment alternative for eligible patients.

### Limitations

As with any economic model, assumptions were used to facilitate this analysis. First, the model relies on real-world mid-term (25 months follow-up) data, which were used to extrapolate clinical outcomes over a 10-year timespan. For more reliable clinical event rates, a longer follow-up would have been preferable, as our knowledge on the implications of both treatments in the long-term remains uncertain. In the absence of long-term comparative data, the assumption of constant event rates over time may not fully capture potential changes in clinical outcomes beyond the observed follow-up. Second, the model did not account for recurrent stroke, despite evidence showing that first-time stroke patients may face a 2.6-fold increased risk of experiencing another stroke [66]. Moreover, we have not considered other clinical outcomes (i.e., hospitalization due to heart failure or falls, cognitive impairment) that may be strictly correlated with AF and may increase healthcare costs. Additionally, treatment discontinuation and suboptimal adherence to DOAC therapy were not considered. Clinical and economic outcomes were modeled under the assumption that patients in the DOAC arm remained adherent over the 10-year horizon. The model also presents limitations related to its input parameters. Given the potential differences in the population and clinical practice, the results of this analysis might be subject to external validity bias. As the analysis was conducted from the perspective of the Italian National Healthcare Service, the applicability of the results to other healthcare settings may be limited by differences in unit cost estimates, which are specific to the Italian context. With respect to cost inputs, as previously noted, the estimated cost of managing peri-procedural complications during LAAO was likely overestimated by the model. This discrepancy arose from using the cost of an index hospitalization for the specific diagnosis as a source of unit cost. However, in cases of peri-procedural complications, the cost is expected to be lower since the patient was already hospitalized to undergo the LAAO procedure. The cost of the LAAO procedure considered in the analysis is an estimation based on an average length of stay and procedure duration; procedure cost in real-world settings is sensitive to the operator experience, volume of LAAO performed, and the ability to optimize the patient pathway.

Additionally, as the budget impact analysis adopted the perspective of the Italian Healthcare System, the focus was exclusively on direct healthcare costs associated with clinical events. In some instances, namely for stroke, the costs related to informal care and productivity losses represent a highly significant burden on lifetime costs [67]. This resulted in a significant underestimation of the economic and societal impact of stroke as described by the model.

Lastly, the LAAO group included only WATCHMAN FLX^™^ devices; therefore, the findings of our study cannot be generalized to other LAAO technologies. Therefore, the findings of our study cannot be directly generalized to other LAAO technologies. Differences in device design, implantation techniques, and procedural costs may influence both clinical and economic outcomes.

## 5. Conclusions

Our budget impact analysis under the Italian Healthcare Service suggests that the expansion of LAAO therapy in a population of non-valvular AF patients with a CHA_2_DS_2_-VASc score ≥ 5 may yield substantial long-term economic benefits, as the initial investment in the LAAO procedure and device would be counterbalanced within 8 years.

## Figures and Tables

**Table 1:** Patient funnel predicting the reference patient cohort. AF: atrial fibrillation; DOAC: direct oral anticoagulation.

Patient Funnel			Source
**Total population**		58,934,177	Report ISTAT [21]
**AF patients’ prevalence**	1.87%	1,100,391	Elaboration on Andreotti et al. [22], based on AF prevalence projections by Di Carlo et al. [23]
**Non-valvular AF**	94.6%	1,040,970	Kirchhof et al. [24]
**CHA_2_DS_2_-VAS_C_ ≥ 5**	22%	229,013	Caterina et al. [25]
**Estimated DOAC population in 2025**	67%	153,439	Bo et al. [26]; Olimpieri et al. [27]

**Table 2:** Cumulative cost over 10 years: breakdown by cost category. LAAO: left atrial appendage occlusion.

Cost Category	Cumulative Cost over 10 Years, €				
	Baseline Scenario	Alternative Scenario 1	*Scenario 1 vs. Baseline*	Alternative Scenario 2	*Scenario 2 vs. Baseline*
LAAO Procedure ^a^	9,533,857	23,560,927	14,027,070	54,544,183	45,010,328
Adverse Events ^b^	676,677,029	672,247,198	−4,429,830	662,462,505	−14,214,523
Long-term Stroke Related ^c^	937,298,632	930,406,575	−6,892,057	915,183,267	−22,115,365
Pharmacological Treatment	610,195,951	602,580,619	−7,615,332	585,759,729	−24,436,222
**Total**	**2,233,705,469**	**2,228,795,319**	**−4,910,150**	**2,217,949,685**	**−15,755,784**

^a^ The cost of the LAAO procedure includes the cost of the device, the cost of the procedure, the hospitalization cost, the cost of procedural complications, and the cost of peri-procedural pharmacological treatment up to 7 days post-implant. ^b^ The cost of adverse events includes the cost of adverse events after discharge (7 days post-implant); for stroke, only the acute phase is considered. ^c^ The long-term stroke cost includes the post-acute management of stroke patients.

**Table 3:** Cumulative cost and n. of events for major bleeding and ischemic stroke (over 10 years).

Clinical Outcomes	Cumulative n. of Events and Related Costs			
	Alternative Scenario 1 vs. Baseline		Alternative Scenario 2 vs. Baseline	
	∆ n. of Clinical Events	∆ Cumulative Related Costs	∆ n. of Clinical Events	∆ Cumulative Related Costs
**Major bleeding**	−324	−€2,502,721	−1040	−€8,030,780
**Ischemic stroke**	−41	−€267,912	−130	−€859,681

## Data Availability

The data underlying this article will be shared on reasonable request to the corresponding author.

## Supplementary Materials

The following supporting information can be downloaded at: https://www.mdpi.com/article/10.3390/jcm15103687/s1, Supplementary Method (Definitions and Statistical Analysis). Supplemental Figure S1. Tornado plot of sensitivity analysis. The x-axis represents the cost savings in Alternative Scenario I versus the baseline at 10 years. Supplemental Figure S2. Tornado plot of sensitivity analysis. The x-axis represents the cost savings in Alternative Scenario II versus the baseline at 10 years. Supplemental Table S1. Cost inputs. Supplemental Table S2. Baseline characteristics. Supplemental Table S3. Periprocedural adverse events among LAAO patients. Supplemental Table S4. Major adverse events during follow-up [68,69,70,71].

## Abbreviations

AF	Atrial Fibrillation
CV	Cardiovascular
DDD	Defined Daily Dose
DOAC	Direct Oral Anticoagulation
GISE	Italian Society of Interventional Cardiology
LAAO	Left Atrial Appendage Occlusion
mRs	Modified Rankin Scale
TE	Thromboembolic
TTD	Time-to-Treatment Discontinuations

## References

[1] Chugh S.S., Havmoeller R., Narayanan K., Singh D., Rienstra M., Benjamin E.J., Gillum R.F., Kim Y.H., McAnulty J.H., Zheng Z.J. (2014). Worldwide Epidemiology of Atrial Fibrillation: A Global Burden of Disease 2010 Study. Circulation.

[2] Van Gelder I.C., Rienstra M., Bunting K.V., Casado-Arroyo R., Caso V., Crijns H.J., De Potter T.J., Dwight J., Guasti L., Hanke T. (2024). 2024 ESC Guidelines for the management of atrial fibrillation developed in collaboration with the European Association for Cardio-Thoracic Surgery (EACTS). Eur. Heart J..

[3] Elsheikh S., Hill A., Irving G., Lip G.Y., Abdul-Rahim A.H. (2024). Atrial fibrillation and stroke: State-of-the-art and future directions. Curr. Probl. Cardiol..

[4] Piccini J.P., Hammill B.G., Sinner M.F., Jensen P.N., Hernandez A.F., Heckbert S.R., Benjamin E.J., Curtis L.H. (2012). Incidence and Prevalence of Atrial Fibrillation and Associated Mortality Among Medicare Beneficiaries: 1993–2007. Circ. Cardiovasc. Qual. Outcomes.

[5] Luengo-Fernandez R., Violato M., Candio P., Leal J. (2020). Economic burden of stroke across Europe: A population-based cost analysis. Eur. Stroke J..

[6] Hindricks G., Potpara T., Dagres N., Arbelo E., Bax J.J., Blomström-Lundqvist C., Boriani G., Castella M., Dan G.A., Dilaveris P.E. (2021). 2020 ESC Guidelines for the diagnosis and management of atrial fibrillation developed in collaboration with the European Association for Cardio-Thoracic Surgery (EACTS). Eur. Heart J..

[7] Lip G.Y., Laroche C., Dan G.A., Santini M., Kalarus Z., Rasmussen L.H., Oliveira M.M., Mairesse G., Crijns H.J., Simantirakis E. (2014). A prospective survey in European Society of Cardiology member countries of atrial fibrillation management: Baseline results of EURObservational Research Programme Atrial Fibrillation (EORP-AF) Pilot General Registry. Europace.

[8] Hsu J.C., Maddox T.M., Kennedy K.F., Katz D.F., Marzec L.N., Lubitz S.A., Gehi A.K., Turakhia M.P., Marcus G.M. (2016). Oral Anticoagulant Therapy Prescription in Patients with Atrial Fibrillation Across the Spectrum of Stroke Risk: Insights From the NCDR PINNACLE Registry. JAMA Cardiol..

[9] O’Brien E.C., Simon D.N., Allen L.A., Singer D.E., Fonarow G.C., Kowey P.R., Thomas L.E., Ezekowitz M.D., Mahaffey K.W., Chang P. (2014). Reasons for warfarin discontinuation in the Outcomes Registry for Better Informed Treatment of Atrial Fibrillation (ORBIT-AF). Am. Heart J..

[10] Magnocavallo M., Bellasi A., Mariani M.V., Fusaro M., Ravera M., Paoletti E., Di Iorio B., Barbera V., Della Rocca D.G., Palumbo R. (2020). Thromboembolic and Bleeding Risk in Atrial Fibrillation Patients with Chronic Kidney Disease: Role of Anticoagulation Therapy. JCM.

[11] Holmes D.R., Kar S., Price M.J., Whisenant B., Sievert H., Doshi S.K., Huber K., Reddy V.Y. (2014). Prospective Randomized Evaluation of the Watchman Left Atrial Appendage Closure Device in Patients with Atrial Fibrillation Versus Long-Term Warfarin Therapy. J. Am. Coll. Cardiol..

[12] Reddy V.Y., Sievert H., Halperin J., Doshi S.K., Buchbinder M., Neuzil P., Huber K., Whisenant B., Kar S., Swarup V. (2014). Percutaneous Left Atrial Appendage Closure vs. Warfarin for Atrial Fibrillation: A Randomized Clinical Trial. JAMA.

[13] Magnocavallo M., Della Rocca D.G., Vetta G., Mohanty S., Gianni C., Polselli M., Rossi P., Parlavecchio A., La Fazia M.V., Guarracini F. (2024). Lower rate of major bleeding in very high risk patients undergoing left atrial appendage occlusion: A propensity score–matched comparison with direct oral anticoagulant. Heart Rhythm..

[14] Mohanty S., Mohanty P., Trivedi C., Assadourian J., Mayedo A.Q., MacDonald B., Della Rocca D.G., Gianni C., Horton R., Al-Ahmad A. (2021). Impact of Oral Anticoagulation Therapy Versus Left Atrial Appendage Occlusion on Cognitive Function and Quality of Life in Patients with Atrial Fibrillation. JAHA.

[15] Osmancik P., Herman D., Neuzil P., Hala P., Taborsky M., Kala P., Poloczek M., Stasek J., Haman L., Branny M. (2020). Left Atrial Appendage Closure Versus Direct Oral Anticoagulants in High-Risk Patients with Atrial Fibrillation. J. Am. Coll. Cardiol..

[16] Reddy V.Y., Möbius-Winkler S., Miller M.A., Neuzil P., Schuler G., Wiebe J., Sick P., Sievert H. (2013). Left atrial appendage closure with the Watchman device in patients with a contraindication for oral anticoagulation: The ASAP study (ASA Plavix Feasibility Study with Watchman Left Atrial Appendage Closure Technology). J. Am. Coll. Cardiol..

[17] Nielsen-Kudsk J.E., Korsholm K., Damgaard D., Valentin J.B., Diener H.C., Camm A.J., Johnsen S.P. (2021). Clinical Outcomes Associated with Left Atrial Appendage Occlusion Versus Direct Oral Anticoagulation in Atrial Fibrillation. JACC Cardiovasc. Interv..

[18] Sullivan S.D., Mauskopf J.A., Augustovski F., Caro J.J., Lee K.M., Minchin M., Orlewska E., Penna P., Barrios J.M.R., Shau W.Y. (2014). Budget Impact Analysis—Principles of Good Practice: Report of the ISPOR 2012 Budget Impact Analysis Good Practice II Task Force. Value Health.

[19] Della Rocca D.G., Magnocavallo M., Di Biase L., Mohanty S., Trivedi C., Tarantino N., Gianni C., Lavalle C., Van Niekerk C.J., Romero J. (2021). Half-Dose Direct Oral Anticoagulation Versus Standard Antithrombotic Therapy After Left Atrial Appendage Occlusion. JACC Cardiovasc. Interv..

[20] Della Rocca D.G., Horton R.P., Di Biase L., Gianni C., Trivedi C., Mohanty S., Anannab A., Magnocavallo M., Chen Q., Tarantino N. (2021). Incidence of Device-Related Thrombosis in Watchman Patients Undergoing a Genotype-Guided Antithrombotic Strategy. JACC Clin. Electrophysiol..

[21] Battaglini M., Simone M. (2024). Indicatori Demografici Anno 2023.

[22] Andreotti F., D’Angela D., Mancusi L., Spandonaro F. (2017). Prevalenza della fibrillazione atriale, eleggibilità al trattamento e consumo di anticoagulanti orali nelle Aziende Sanitarie italiane: Impatto dei nuovi anticoagulanti. G. Ital. Di Cardiol..

[23] Di Carlo A., Bellino L., Consoli D., Mori F., Zaninelli A., Baldereschi M., Cattarinussi A., D’Alfonso M.G., Gradia C., Sgherzi B. (2019). Prevalence of atrial fibrillation in the Italian elderly population and projections from 2020 to 2060 for Italy and the European Union: The FAI Project. EP Eur..

[24] Kirchhof P., Ammentorp B., Darius H., De Caterina R., Le Heuzey J.Y., Schilling R.J., Schmitt J., Zamorano J.L. (2014). Management of atrial fibrillation in seven European countries after the publication of the 2010 ESC Guidelines on atrial fibrillation: Primary results of the PREvention oF thromboemolic events—European Registry in Atrial Fibrillation (PREFER in AF). EP Eur..

[25] De Caterina R., Renda G., Sangiuolo R., Attena E., Di Lecce L., Romeo F. (2014). La gestione del rischio tromboembolico nei pazienti con fibrillazione atriale in Italia: Dati al basale del Registro Europeo PREFER in AF. G. Ital. Cardiol..

[26] Bo M., Fumagalli S., Degli Esposti L., Perrone V., Dovizio M., Poli D., Marcucci R., Verdecchia P., Reboldi G., Lip G.Y.H. (2024). Anticoagulation in atrial fibrillation. A large real-world update. Eur. J. Intern. Med..

[27] Olimpieri P.P., Di Lenarda A., Mammarella F., Gozzo L., Cirilli A., Cuomo M., Gulizia M.M., Colivicchi F., Murri G., Gabrielli D. (2020). Non-vitamin K antagonist oral anticoagulation agents in patients with atrial fibrillation: Insights from Italian monitoring registries. IJC Heart Vasc..

[28] Musto C. (2025). Dati di Attività Delle Cardiologia Interventistica GISE 2024—Interventistica Strutturale.

[29] Zeitler E.P., Bian B., Griffiths R.I., Allocco D.J., Christen T., Roy K., Cohen D.J., Reynolds M.R. (2024). Long-Term Clinical Outcomes Following the WATCHMAN Device Use in Medicare Beneficiaries. Circ. Cardiovasc. Qual. Outcomes.

[30] Maarse M., Aarnink E.W., Huijboom M.F., Abeln B.G., Staal D., Rensing B.J., Kerklaan J.P., van Dijk V.F., Swaans M.J., Boersma L.V. (2023). Long-term outcomes of successful left atrial appendage occlusion with focus on stroke prevention: 10-year follow-up of a single-center registry. Heart Rhythm O2.

[31] Kar S., Doshi S.K., Sadhu A., Horton R., Osorio J., Ellis C., Stone J., Shah M., Dukkipati S.R., Adler S. (2021). Primary Outcome Evaluation of a Next-Generation Left Atrial Appendage Closure Device: Results from the PINNACLE FLX Trial. Circulation.

[32] van Swieten J.C., Koudstaal P.J., Visser M.C., Schouten H., Van Gijn J. (1988). Interobserver agreement for the assessment of handicap in stroke patients. Stroke.

[33] Berti S., De Caterina A.R., Grasso C., Casu G., Giacchi G., Pagnotta P., Maremmani M., Mazzone P., Limite L., Tomassini F. (2023). Periprocedural outcome in patients undergoing left atrial appendage occlusion with the Watchman FLX device: The ITALIAN-FLX registry. Front. Cardiovasc. Med..

[34] Galea R., De Marco F., Meneveau N., Aminian A., Anselme F., Gräni C., Huber A.T., Teiger E., Iriart X., Babongo Bosombo F. (2022). Amulet or Watchman Device for Percutaneous Left Atrial Appendage Closure: Primary Results of the SWISS-APERO Randomized Clinical Trial. Circulation.

[35] Fattore G., Torbica A., Susi A., Giovanni A., Benelli G., Gozzo M., Toso V. (2012). The social and economic burden of stroke survivors in Italy: A prospective, incidence-based, multi-centre cost of illness study. BMC Neurol..

[36] Jommi C., Iorio A., Crippa L., Gelera A., Senatore G. (2013). Analisi di impatto sul budget di WatchmanTM, dispositivo per la prevenzione tromboembolica nei pazienti con fibrillazione atriale. PharmacoEconomics Ital. Res. Artic..

[37] Pradelli L., Calandriello M., Di Virgilio R., Bellone M., Tubaro M. (2014). Budget impact analysis of apixaban versus other NOACs for the prevention of stroke in Italian atrial fibrillation patients. Farmeconomia.

[38] Rivaluta (2025). Rivalutazioni e Documentazione su Prezzi, Costi e Retribuzioni Contrattuali.

[39] Lip G.Y., Lane D.A. (2015). Stroke Prevention in Atrial Fibrillation: A Systematic Review. JAMA.

[40] Mariani M.V., Magnocavallo M., Straito M., Piro A., Severino P., Iannucci G., Chimenti C., Mancone M., Rocca D.G.D., Forleo G.B. (2021). Direct oral anticoagulants versus vitamin K antagonists in patients with atrial fibrillation and cancer a meta-analysis. J. Thromb. Thrombolysis.

[41] Hutt E., Wazni O.M., Kaur S., Saliba W.I., Tarakji K.G., Kapadia S., Aguilera J., Barakat A.F., Abdallah M., Jaber W. (2020). Left atrial appendage closure device implantation in patients at very high risk for stroke. Heart Rhythm..

[42] Camm A.J., Cools F., Virdone S., Bassand J.P., Fitzmaurice D.A., Arthur Fox K.A., Goldhaber S.Z., Goto S., Haas S., Mantovani L.G. (2020). Mortality in Patients with Atrial Fibrillation Receiving Nonrecommended Doses of Direct Oral Anticoagulants. J. Am. Coll. Cardiol..

[43] Nielsen P.B., Skjøth F., Søgaard M., Kjældgaard J.N., Lip G.Y., Larsen T.B. (2017). Effectiveness and safety of reduced dose non-vitamin K antagonist oral anticoagulants and warfarin in patients with atrial fibrillation: Propensity weighted nationwide cohort study. BMJ.

[44] Steinberg B.A., Shrader P., Thomas L., Ansell J., Fonarow G.C., Gersh B.J., Kowey P.R., Mahaffey K.W., Naccarelli G., Reiffel J. (2016). Off-Label Dosing of Non-Vitamin K Antagonist Oral Anticoagulants and Adverse Outcomes. J. Am. Coll. Cardiol..

[45] Yao X., Shah N.D., Sangaralingham L.R., Gersh B.J., Noseworthy P.A. (2017). Non–Vitamin K Antagonist Oral Anticoagulant Dosing in Patients with Atrial Fibrillation and Renal Dysfunction. J. Am. Coll. Cardiol..

[46] Osmancik P., Herman D., Neuzil P., Hala P., Taborsky M., Kala P., Poloczek M., Stasek J., Haman L., Branny M. (2022). 4-Year Outcomes After Left Atrial Appendage Closure Versus Nonwarfarin Oral Anticoagulation for Atrial Fibrillation. J. Am. Coll. Cardiol..

[47] Saliba W., Nair D., Swarup V., Hall T., Iyer V., Pérez G.C., Weiner S., Shah M., Islam N., Grygier M. (2025). Comparison of left atrial appendage closure and oral anti-coagulation after catheter ablation for atrial fibrillation: Concomitant and sequential cohorts of the OPTION randomized controlled trial. Heart Rhythm..

[48] Turagam M.K., Kawamura I., Neuzil P., Nair D., Doshi S., Valderrabano M., Hala P., Della Rocca D., Gibson D., Funasako M. (2024). Ischemic stroke severity and mortality after left atrial appendage closure vs. nonwarfarin oral anticoagulants in patients with prior stroke. Heart Rhythm..

[49] The Medicines Utilisation Monitoring Centre (2019). National Report on Medicines Use in Italy—2018.

[50] Schmidt B., Bordignon S., Neven K., Reichlin T., Blaauw Y., Hansen J., Adelino R., Ouss A., Füting A., Roten L. (2023). EUropean real-world outcomes with Pulsed field ablatiOn in patients with symptomatic atRIAl fibrillation: Lessons from the multi-centre EU-PORIA registry. Europace.

[51] Della Rocca D.G., Sorgente A., Pannone L., Cespón-Fernández M., Vetta G., Almorad A., Bala G., Del Monte A., Ströker E., Sieira J. (2024). Multielectrode catheter-based pulsed field ablation of persistent and long-standing persistent atrial fibrillation. Europace.

[52] Tam M.T., Kojodjojo P., Lam Y.Y., Chow J., Wong C., Kam K.K.H., Wong G.L.N., Chan C.P., Chan J.Y., So K.C.Y. (2025). Combined pulsed field ablation and left atrial appendage occlusion: A multicenter comparative study. Heart Rhythm..

[53] Ekanem E., Reddy V.Y., Schmidt B., Reichlin T., Neven K., Metzner A., Hansen J., Blaauw Y., Maury P., Arentz T. (2022). Multi-national survey on the methods, efficacy, and safety on the post-approval clinical use of pulsed field ablation (MANIFEST-PF). Europace.

[54] Della Rocca D.G., Marcon L., Magnocavallo M., Menè R., Pannone L., Mohanty S., Sousonis V., Sorgente A., Almorad A., Bisignani A. (2023). Pulsed electric field, cryoballoon, and radiofrequency for paroxysmal atrial fibrillation ablation: A propensity score-matched comparison. Europace.

[55] Wazni O.M., Boersma L., Healey J.S., Mansour M., Tondo C., Phillips K., Doshi R., Jaber W., Hynes E., Allocco D.J. (2022). Comparison of anticoagulation with left atrial appendage closure after atrial fibrillation ablation: Rationale and design of the OPTION randomized trial. Am. Heart J..

[56] Amorosi S.L., Armstrong S., Da Deppo L., Garfield S., Stein K. (2014). The budget impact of left atrial appendage closure compared with adjusted-dose warfarin and dabigatran etexilate for stroke prevention in atrial fibrillation. Europace.

[57] Panikker S., Lord J., Jarman J.W., Armstrong S., Jones D.G., Haldar S., Butcher C., Khan H., Mantziari L., Nicol E. (2016). Outcomes and costs of left atrial appendage closure from randomized controlled trial and real-world experience relative to oral anticoagulation. Eur. Heart J..

[58] Singh S.M., Micieli A., Wijeysundera H.C. (2013). Economic Evaluation of Percutaneous Left Atrial Appendage Occlusion, Dabigatran, and Warfarin for Stroke Prevention in Patients with Nonvalvular Atrial Fibrillation. Circulation.

[59] Reddy V.Y., Akehurst R.L., Armstrong S.O., Amorosi S.L., Beard S.M., Holmes D.R. (2015). Time to Cost-Effectiveness Following Stroke Reduction Strategies in AF. J. Am. Coll. Cardiol..

[60] Reddy V.Y., Akehurst R.L., Amorosi S.L., Gavaghan M.B., Hertz D.S., Holmes D.R. (2018). Cost-Effectiveness of Left Atrial Appendage Closure with the WATCHMAN Device Compared with Warfarin or Non–Vitamin K Antagonist Oral Anticoagulants for Secondary Prevention in Nonvalvular Atrial Fibrillation. Stroke.

[61] Reddy V.Y., Akehurst R.L., Gavaghan M.B., Amorosi S.L., Holmes D.R. (2019). Cost-Effectiveness of Left Atrial Appendage Closure for Stroke Reduction in Atrial Fibrillation: Analysis of Pooled, 5-Year, Long-Term Data. JAHA.

[62] Freeman J.V., Hutton D.W., Barnes G.D., Zhu R.P., Owens D.K., Garber A.M., Go A.S., Hlatky M.A., Heidenreich P.A., Wang P.J. (2016). Cost-Effectiveness of Percutaneous Closure of the Left Atrial Appendage in Atrial Fibrillation Based on Results from PROTECT AF Versus PREVAIL. Circ. Arrhythmia Electrophysiol..

[63] Lee V.W.Y., Tsai R.B.C., Chow I.H.I., Yan B.P.Y., Kaya M.G., Park J.W., Lam Y.Y. (2016). Cost-effectiveness analysis of left atrial appendage occlusion compared with pharmacological strategies for stroke prevention in atrial fibrillation. BMC Cardiovasc. Disord..

[64] Kawakami H., Nolan M.T., Phillips K., Scuffham P.A., Marwick T.H. (2021). Cost-effectiveness of combined catheter ablation and left atrial appendage closure for symptomatic atrial fibrillation in patients with high stroke and bleeding risk. Am. Heart J..

[65] Labori F., Persson J., Bonander C., Jood K., Svensson M. (2022). Cost-effectiveness analysis of left atrial appendage occlusion in patients with atrial fibrillation and contraindication to oral anticoagulation. Eur. Heart J..

[66] (1996). Stroke Prevention in Atrial Fibrillation Investigators. Adjusted-dose warfarin versus low-intensity, fixed-dose warfarin plus aspirin for high-risk patients with atrial fibrillation: Stroke Prevention in Atrial Fibrillation III randomised clinical trial. Lancet.

[67] Truelsen T., Ekman M., Boysen G. (2005). Cost of stroke in Europe. Euro. J. Neurol..

[68] Tzikas A., Holmes D.R., Gafoor S., Ruiz C.E., Blomström-Lundqvist C., Diener H., Cappato R., Kar S., Lee R.J., Byrne R.A. (2017). Percutaneous left atrial appendage occlusion: The Munich consensus document on definitions, endpoints, and data collection requirements for clinical studies. Europace.

[69] Schulman S., Kearon C. (2005). Definition of major bleeding in clinical investigations of antihemostatic medicinal products in non-surgical patients. J. Thromb. Haemost..

[70] Thygesen K., Alpert J.S., Jaffe A.S., Chaitman B.R., Bax J.J., Morrow D.A., White H.D., Executive Group on behalf of the Joint European Society of Cardiology (ESC)/American College of Cardiology (ACC)/American Heart Association (AHA)/World Heart Federation (WHF) Task Force for the Universal Definition of Myocardial Infarction (2019). Fourth universal definition of myocardial infarction (2018). Eur. Heart J..

[71] Berti E., Fortuna D., Bartoli S., Ciuca C., Orlando A., Scondotto S., Agabiti N., Salizzoni S., Aranzulla T.C., Gandolfo C. (2016). I costi di ricovero e follow-up delle procedure di sostituzione valvolare aortica per via percutanea e cardiochirurgica a confronto: Analisi secondo le prospettive del Sistema Sanitario Regionale e dell’Ospedale. G. Ital. Cardiol..

