# Supplementary material for: Left Atrial Appendage Occlusion Versus Direct Oral Anticoagulation in Atrial Fibrillation Patients at Very High Risk of Stroke: A Budget Impact Analysis in Italy
Source: J Clin Med. 2026 May 11;15(10):3687. doi: 10.3390/jcm15103687 (PMC13207641; doi:10.3390/jcm15103687)
Supplement: Supplementary file 1 [file jcm-15-03687-s001.zip › jcm-4233050-supplementary.pdf]

SUPPLEMENTAL MATERIALS

Left Atrial Appendage Occlusion versus Direct Oral Anticoagulation for Stroke Prevention in Atrial Fibrillation: A Budget Impact Analysis in Italy

Supplemental Material

Definitions.

Statistical Analysis.

Additional Figures

Supplemental Figure S1.

Supplemental Figure S2.

Additional Tables

Supplemental Table S1.

Supplemental Table S2.

Supplemental Table S3.

Supplemental Table S4.

References

Supplemental Material

Definitions.

Thromboembolic events were defined according to the Munich consensus[69]. Bleeding events were categorized according to the International Society of Thrombosis and Hemostasis[70]. Major bleeding included: fatal bleeding, symptomatic bleeding in a critical area or organ (e.g., intracranial), and/or bleeding causing a fall in hemoglobin ≥2g/dL or requiring transfusion of ≥2 units of packed red blood cells or whole blood. Clinically relevant minor bleeding included bleeding requiring a clinical response, such as hospital admission, physician guided medical or surgical treatment, or need for antithrombotic therapy changes. Non-clinically relevant minor bleeding was defined as all reported bleedings not classified as major or clinically relevant minor.

For the purpose of the study, all bleeding events requiring hospital admission were considered in the cost assessment.

Myocardial infarction was defined as an elevation of the cardiac troponin values with at least one value above the 99th percentile upper reference limit; the myocardial injury is considered acute if there is a rise and/or fall of troponin values[71].

In patients undergoing LAAO, major peri-procedural adverse events included death from any cause, stroke, TIA, peripheral embolism, clinically relevant bleeding, myocardial infarction, device embolization, acute heart failure, pericardial effusion requiring surgery or percutaneous drainage, retroperitoneal hematoma or other major vascular complications requiring surgical repair, occurring within 7 days after the procedure.

Statistical Analysis.

The normal distribution of all continuous variables was checked by visual methods (Q-Q plot and histogram) and the significance test (Kolmogorov-Smirnov normality test and Shapiro-Wilk's test). For continuous variables, descriptive statistics were provided (number of available observations, mean, standard deviation), while median [interquartile range (IQR)] was used for non-normal data.

Categorical data were described as simple frequency and percentage (%). Student's t-test, the $\chi 2$ test, and the Fisher exact test were used for comparisons.

Propensity score matching was performed to reduce the risk of selection bias. Patients were divided into 2 cohorts: patients treated with Watchman FLX™ (Boston Scientific Corporation, Marlborough, MA, USA) implantation and patients treated with DOACs. The propensity score is defined as the subject's probability of receiving a specific treatment or exposure given a set of measured baseline covariates. The model considered each single covariate of the CHA_2DS_2-VASc (congestive heart failure, hypertension, age ≥ 75 years, diabetes mellitus, prior stroke or transient ischemic attack or thromboembolism, vascular disease, age 65–74 years, sex category) and HAS-BLED (hypertension, abnormal renal or liver function, stroke, bleeding, labile international normalized ratio, elderly, drugs or alcohol) score. Matching was performed using the nearest neighbor matching protocol (matching ratio of 1 to 2 with-out replacement) and a caliper width of 0.01. The balance of characteristics was assessed by estimating standardized differences between groups; standardized difference indicates the degree of systematic differences in covariates between groups. Operationally, a standardized difference >10% represents a meaningful imbalance in a given variable between groups. For all tests, a p-value <0.05 was considered statistically significant. To assess the robustness of our budget impact model, we conducted a one-way sensitivity analysis by varying all model parameters to measure their impact on the key budgetary outcomes. All input data are varied according to their 95% confidence intervals (CIs), or by applying a ±10% range of variation for input values where the 95% CI is unavailable.

Supplemental Figure S1. Tornado plot of sensitivity analysis. The x-axis represents the cost savings in Alternative Scenario I versus Baseline at 10 years. All variables impacting savings by more than 5% displayed. *DOAC: Direct Oral Anticoagulant; LAAO: Left Atrial Appendage Occlusion; TIA: Transient Ischemic Attack.*

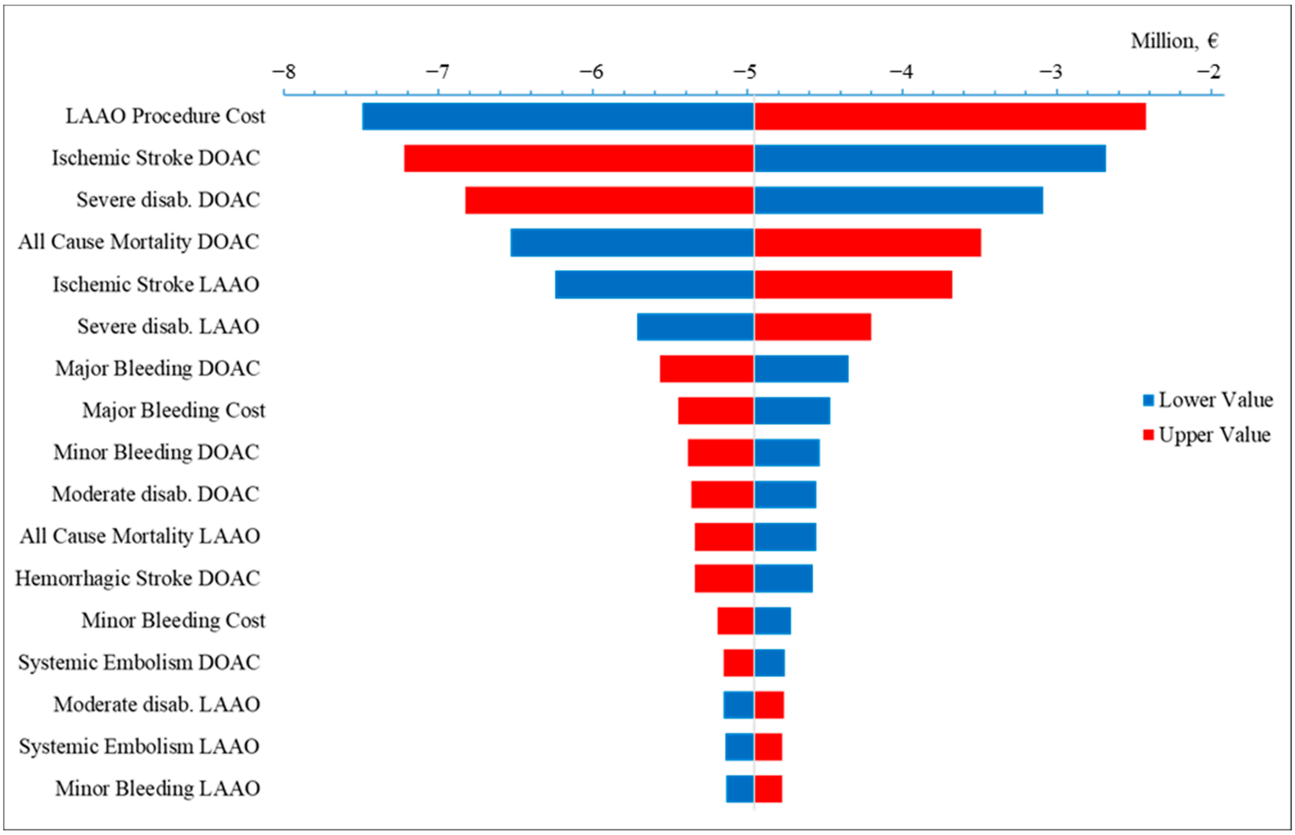

Supplemental Figure S2. Tornado plot of sensitivity analysis. The x-axis represents the cost savings in Alternative Scenario II versus Baseline at 10 years. All variables impacting savings by more than 5% displayed. *DOAC: Direct Oral Anticoagulation; LAAO: Left Atrial Appendage Occlusion; TIA: Transient Ischemic Attack.*

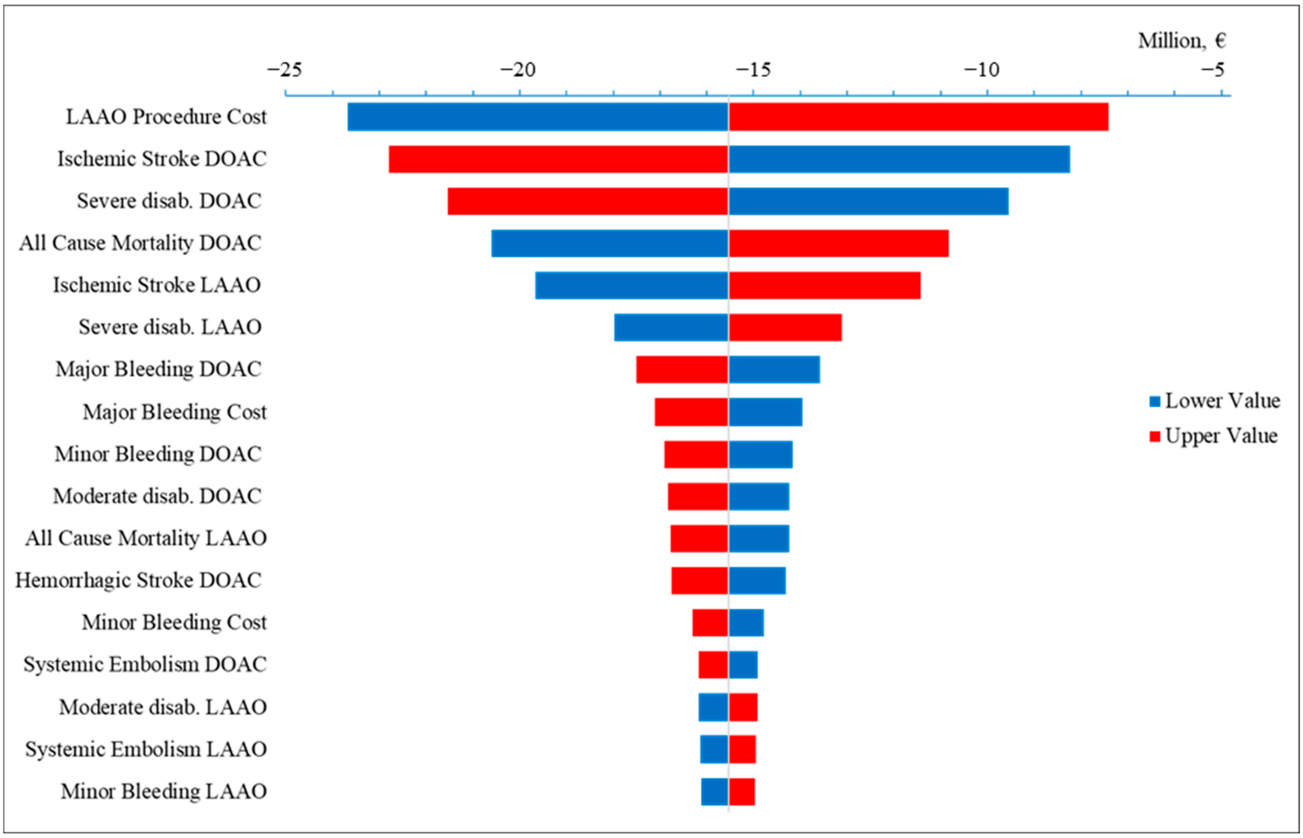

Supplemental Table S1. Cost inputs. *ASA: Acetylsalicylic Acid; DDD: Defined Daily Dose; DRG: Diagnosis-related Group; LAAO: Left Atrial Appendage Occlusion; TIA: Transient Ischemic Attack.*

Event	Unit Cost	Source
**LAAO procedure and peri-procedural complications**		
LAAO Procedure and Hospitalization	€ 6,555.18	Cath lab:<br>Berti E et al., 2016[72];<br><br>Human resources:<br>Average per capita salary by category of public administration employees, Ministry of Economy and Finance (2021)[a]<br><br>Hospitalization:<br>Berti E et al., 2016 [72];
Pericardial Effusion	€ 10,208.98	Jommi et al., 2013 [30]
Femoral pseudoaneurysm	€ 1,090.00	DRG 131 "Malattie vascolari periferiche senza CC"
Ischemic stroke	€ 7,623.38	Fattore et al., 2013 [29]
Major bleeding	€ 7,720.45	Jommi et al., 2013 [30]
Minor bleeding	€ 4,591.65	Elaboration[b] on Jommi et al., 2013 [30]
**Adverse events**		
Ischemic stroke	€ 7,623.38	Fattore et al., 2013 [29]
Hemorrhagic Stroke	€ 8,917.07	Fattore et al., 2013 [29]
Myocardial Infarction	€ 8,646.95	Pradelli et al., 2014 [31]
Major bleeding	€ 7,720.45	Jommi et al., 2013 [30]
Minor bleeding	€ 4,591.65	Elaboration[b] on Jommi et al., 2013 [30]
Systemic embolism	€ 10,343.60	Jommi et al., 2013 [30]
TIA	€ 2,543.00	DRG 524 " ischemia cerebrale transitoria"
**Long-term Stroke management (post-acute) – yearly cost**		
All-stroke – No/mild disability (mRS 0−2)	€ 2,707.49	Fattore et al., 2013 [29]
All-stroke – Moderate disability (mRS 3)	€ 7,219.62	Fattore et al., 2013 [29]
All-stroke – Severe disability (mRS 4−5)	€ 18,439.71	Fattore et al., 2013 [29]
**Pharmacological treatment – daily cost**		
Dual Antiplatlet Therapy: Aspirin + Clopidogrel	€ 0.24	codifa.it, Clopidogrel + ASA (DDD:1 x 75 mg clopidogrel + 100 mg ASA)

VKA	€ 0.48	Codifa.it; Coumadin (DDD: 1 x 5mg tablet)
INR monitoring	€ 0.70	Blood draw and prothrombin 2 x month + general check-up 4 x year
Aspirin	€ 0.03	codifa.it, 04/10/2023; ASA (DDD: 1 x 100 mg)
Dabigatran	€ 0.83	AIFA[c] (DDD: 2 x 150 mg tablet). Cost of generic drug
Rivaroxaban	€ 0.61	AIFA[d] (DDD: 1 x 20 mg tablet). Cost of generic drug
Apixaban	€ 2.35	AIFA[e] Eliquis (DDD: 2 x 5 mg tablet)
Edoxaban	€ 2.32	AIFA[f] Lixiana (DDD: 1 x 30 mg tablet)

a. https://contoannuale.rgs.mef.gov.it/web/sicosito/spese-e-retribuzioni/retribuzione-media-acc

b. For unit cost of minor bleedings events, due to lack of information the same ratio of minor bleeding tariffs over major bleeding tariffs observed in the Italian DRG reimbursement system (i.e., €3.193 for major bleeding based on DRG 174 "Emorragia gastrointestinale con CC"; €1.899 for minor bleeding based on DRG 175 "Emorragia gastrointestinale senza CC") is applied to the cost of major bleedings retrieved from Fattore et al., 2013[6].

c. Gazzetta Ufficiale della Repubblica Italiana. Available from:
https://www.gazzettaufficiale.it/atto/serie_generale/caricaDettaglioAtto/originario?atto.dataPubblicazioneGazzetta=2025-01-27&atto.codiceRedazionale=25A00363;
https://www.gazzettaufficiale.it/atto/serie_generale/caricaDettaglioAtto/originario?atto.dataPubblicazioneGazzetta=2024-06-19&atto.codiceRedazionale=24A03141;
https://www.gazzettaufficiale.it/atto/serie_generale/caricaDettaglioAtto/originario?atto.dataPubblicazioneGazzetta=2024-08-20&atto.codiceRedazionale=24A04235

d. Gazzetta Ufficiale della Repubblica Italiana. Available from:
https://www.gazzettaufficiale.it/atto/serie_generale/caricaDettaglioAtto/originario?atto.dataPubblicazioneGazzetta=2023-06-09&atto.codiceRedazionale=23A03277
https://www.gazzettaufficiale.it/atto/serie_generale/caricaDettaglioAtto/originario?atto.dataPubblicazioneGazzetta=2024-12-06&atto.codiceRedazionale=24A06438

e. Gazzetta Ufficiale della Repubblica Italiana. Available from:
https://www.gazzettaufficiale.it/atto/serie_generale/caricaDettaglioAtto/originario?atto.dataPubblicazioneGazzetta=2024-09-20&atto.codiceRedazionale=24A04845&elenco30giorni=true

f. Gazzetta Ufficiale della Repubblica Italiana. Available from:
https://www.gazzettaufficiale.it/eli/id/2024/03/27/24A01585/SG

Supplemental Table S2. Baseline Characteristics. *BMI: Body Mass Index, CHF: Congestive Heart Failure, CrCl: Creatinine Clearance; INR: International Normalized Ratio; LAAO: Left Atrial Appendage Occlusion; LVEF: Left Ventricular Ejection Fraction, DOAC: Direct Oral Anticoagulant; SE: Systemic Embolism; TIA: Transient Ischemic Attack.*

	Unmatched			Matched		
	DOAC (n= 494)	LAAO (n= 277)	p-value	DOAC (n= 277)	LAAO (n= 277)	p-value
Clinical characteristics						
**Age, yrs**	80.2 ± 6.6	78.5 ± 7.0	**0.001**	79.6 ± 6.8	78.5 ± 7.0	0.07
**Female**	318 (64.4)	150 (54.2)	**0.006**	168 (60.6)	150 (54.2)	0.14
**Race Black**	9 (1.8)	4 (1.4)	0.78	4 (1.4)	4 (1.4)	1
CHA_2DS_2-VASc Score						
**Mean [range]**	6.0 ± 1 [5-9]	5.8 ± 0.9 [5-9]	**0.001**	5.8 ± 0.9 [5-9]	5.8 ± 0.9 [5-9]	0.74
**Score**						
**5**	188 (38.1)	131 (47.3)	**0.01**	129 (46.6)	131 (47.3)	0.93
**6**	166 (33.6)	90 (32.5)	0.81	89 (32.1)	90 (32.5)	1
**≥7**	140 (28.3)	56 (20.2)	**0.02**	59 (21.3)	56 (20.2)	0.83
HAS-BLED Score						
**Mean [range]**	3.2 ± 0.9 [1-7]	3.0 ± 0.8 [2-6]	**0.002**	3.0 ± 0.9 [2–5]	3.0 ± 0.8 [2-6]	0.80
**Score**						
**2**	143 (28.9)	79 (28.5)	0.93	89 (32.1)	79 (28.5)	0.41
**3**	180 (36.4)	134 (48.4)	**0.001**	119 (43)	134 (48.4)	0.23
**4**	121 (24.5)	50 (18.1)	**0.04**	54 (19.5)	50 (18.1)	0.74
**≥5**	50 (10.2)	14 (5)	**0.02**	15 (5.4)	14 (5)	1
Risk Factors for Stroke and Bleeding						
**CHF**	271 (54.9)	130 (46.9)	**0.03**	149 (53.8)	130 (46.9)	0.11
**Hypertension**	478 (97.4)	261 (94.2)	**0.03**	266 (96)	261 (94.2)	0.43
**Age ≥75**	408 (82.6)	209 (75.5)	**0.02**	225 (81.2)	209 (75.5)	0.12
**Age 65-74**	77 (15.6)	52 (18.8)	0.27	45 (16.2)	52 (18.8)	0.50
**Diabetes Mellitus**	223 (45.1)	107 (38.6)	0.08	114 (41.2)	107 (38.6)	0.60
**Hx. Of Stroke/TIA/SE**	230 (46.6)	141 (50.9)	0.26	126 (45.5)	141 (50.9)	0.24
**Vascular Disease**	326 (66.0)	162 (55.2)	**0.04**	180 (65)	162 (55.2)	0.12
**Abnormal Liver Function**	11 (2.2)	7 (2.5)	0.81	6 (2.2)	7 (2.5)	1
**Abnormal Renal Function**	6 (1.2)	4 (1.4)	1	3 (1.1)	4 (1.4)	1
**Hx. of Bleeding**	153 (31.0)	112 (40.4)	**0.01**	107 (38.6)	112 (40.4)	0.73

**Labile INR**	90 (18.2)	46 (16.6)	0.62	51 (18.4)	46 (16.6)	0.65
**Drug Interactions**	166 (33.6)	121 (43.7)	**0.01**	107 (38.6)	121 (43.7)	0.26
**Alcohol abuse**	14 (2.8)	13 (4.7)	0.22	2 (0.7)	7 (2.5)	0.18

Supplemental Table S3. Periprocedural Adverse Events Among LAAO Patients. *LAAO: Left Atrial Appendage Occlusion; MI: Myocardial Infarction, TIA: Transient Ischemic Attack.*

Procedural Data	LAAO (n= 277)
**Watchman type**	
**2.5**	175 (63.2)
**FLX**	102 (36.8)
**Procedural Duration, min**	88 ± 42
**Contrast, ml**	93 ± 59
**Hospital length of stay, d**	1 [1 – 1]
Device- and Procedural-Related Events	
**Death**	0
**Stroke**	0
**TIA**	1 (0.4)
**Air Embolism**	0
**MI**	0
**Clinically Relevant Bleeding**	1 (0.4)
**Device Embolization**	0
**Respiratory Failure**	0
**Pericardial Effusion**	4 (1.4)
**Requiring Surgery**	1 (0.4)
**Requiring Percutaneous Drainage**	2 (0.7)
**No intervention**	1 (0.4)
**Vascular Complication**	2 (0.7)
**Retroperitoneal Hematoma**	0
**Groin Hematoma**	2 (0.7)
Composite Major Adverse Events	**5 (1.8)**
Overall Adverse Events	**8 (2.9)**

Supplemental Table S4. Major Adverse Events during Follow-Up. *CV: Cardiovascular; GI: Gastrointestinal; IC: Intracranial; mRS: modified Rankin Score; SE: Systemic Embolism; TE: Thromboembolic; TIA: Transient Ischemic Attack.*

Outcome	LAAO (n=277)			DOAC (n=277)			P-value
	Patients with Events	Events	Events/ 100 p-y	Patients with Events	Events	Events/ 100 p-y	
**TE Events**	18 (6.5)	18	3.2	22 (7.9)	22	4.1	0.63
**Stroke/TIA**	15 (5.4)	15	2.6	19 (6.9)	19	3.4	0.59
Disability post Stroke							
No-mild	9 (3.2)	9	1.5	5 (1.8)	5	0.9	0.42
Moderate	2 (0.7)	2	0.4	4 (1.4)	4	0.7	0.69
Severe	3 (1.1)	3	0.6	7 (2.5)	7	1.3	0.34
Fatal Stroke	1 (0.4)	1	0.2	3 (1.1)	3	0.5	0.62
**Major Bleeding**	6 (2.2)	6	1.1	17 (6.1)	17	2.9	**0.03**
**Minor Bleeding**	12 (4.3)	13	2.1	16 (5.8)	21	3.4	0.56
**Follow-up, months**	**25 ± 6**			**26 ± 7**			**0.48**
